# Targeting CD38-Expressing Multiple Myeloma and Burkitt Lymphoma Cells In Vitro with Nanobody-Based Chimeric Antigen Receptors (Nb-CARs)

**DOI:** 10.3390/cells9020321

**Published:** 2020-01-29

**Authors:** Julia Hambach, Kristoffer Riecken, Sophia Cichutek, Kerstin Schütze, Birte Albrecht, Katharina Petry, Jana Larissa Röckendorf, Natalie Baum, Nicolaus Kröger, Timon Hansen, Gunter Schuch, Friedrich Haag, Gerhard Adam, Boris Fehse, Peter Bannas, Friedrich Koch-Nolte

**Affiliations:** 1 Institute of Immunology, University Medical Center Hamburg-Eppendorf (UKE), 20246 Hamburg, Germany; j.hambach@uke.de (J.H.); ke.schuetze@uke.de (K.S.); bi.albrecht@uke.de (B.A.); k.petry@uke.de (K.P.); Jana.Roeckendorf@stud.uke.uni-hamburg.de (J.L.R.); n.baum@uke.de (N.B.); haag@uke.de (F.H.); 2 Department of Diagnostic and Interventional Radiology and Nuclear Medicine, UKE, 20246 Hamburg, Germany; g.adam@uke.de; 3 Research Department Cell and Gene Therapy, UKE, 20246 Hamburg, Germany; k.riecken@uke.uni-hamburg.de (K.R.); sophiac@gmx.net (S.C.); 4 Department of Stem Cell Transplantation, UKE, 20246 Hamburg, Germany; n.kroeger@uke.de; 5 Hematological-Oncology Center Altona, 22767 Hamburg, Germany; Timon.Hansen@hopa-hamburg.de (T.H.); gunter.schuch@hopa-hamburg.de (G.S.)

**Keywords:** CD38, nanobody, chimeric antigen receptor, NK-92 cells, cellular cytotoxicity assays, luciferase, heavy chain antibody, multiple myeloma

## Abstract

The NAD-hydrolyzing ecto-enzyme CD38 is overexpressed by multiple myeloma and other hematological malignancies. We recently generated CD38-specific nanobodies, single immunoglobulin variable domains derived from heavy-chain antibodies naturally occurring in llamas. Nanobodies exhibit high solubility and stability, allowing easy reformatting into recombinant fusion proteins. Here we explore the utility of CD38-specific nanobodies as ligands for nanobody-based chimeric antigen receptors (Nb-CARs). We cloned retroviral expression vectors for CD38-specific Nb-CARs. The human natural killer cell line NK-92 was transduced to stably express these Nb-CARs. As target cells we used CD38-expressing as well as CRISPR/Cas9-generated CD38-deficient tumor cell lines (CA-46, LP-1, and Daudi) transduced with firefly luciferase. With these effector and target cells we established luminescence and flow-cytometry CAR-dependent cellular cytotoxicity assays (CARDCCs). Finally, the cytotoxic efficacy of Nb-CAR NK-92 cells was tested on primary patient-derived CD38-expressing multiple myeloma cells. NK-92 cells expressing CD38-specific Nb-CARs specifically lysed CD38-expressing but not CD38-deficient tumor cell lines. Moreover, the Nb-CAR-NK cells effectively depleted CD38-expressing multiple myeloma cells in primary human bone marrow samples. Our results demonstrate efficacy of Nb-CARs in vitro. The potential clinical efficacy of Nb-CARs in vivo remains to be evaluated.

## 1. Introduction

**Figure 1:**
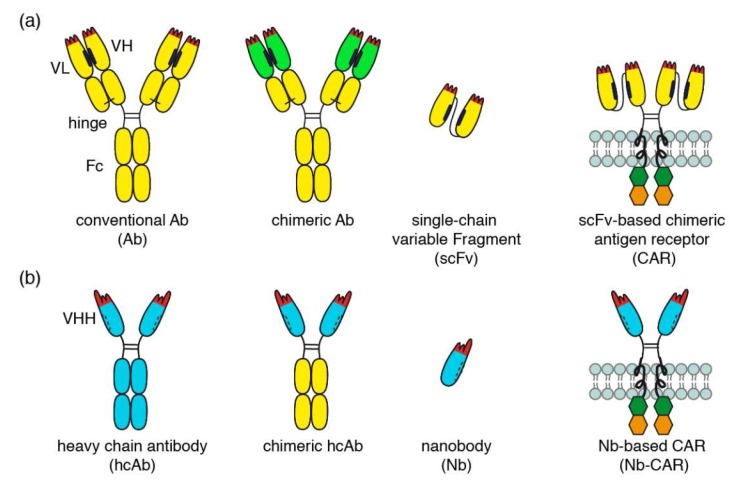
Comparison of conventional and nanobody-based chimeric antigen receptors. (**a**) Schematic diagrams of a conventional antibody (Ab), a chimeric antibody, a single chain variable fragment (scFv), and conventional scFv-based chimeric antigen receptor (CAR). Examples of conventional and chimeric CD38-specific Abs include daratumumab and MOR202 (conventional Abs) and isatuximab (chimeric Ab). (**b**) Schematic diagrams of a camelid heavy chain antibody (hcAb), a chimeric hcAb, a nanobody (Nb) and a Nb-based chimeric antigen receptor (Nb-CAR).

The NAD-hydrolyzing ecto-enzyme CD38 is overexpressed by multiple myeloma and other hematological malignancies [1,2,3]. CD38 has emerged as a promising target for therapy with cytotoxic antibodies. Three monoclonal antibodies have recently entered the clinic (daratumumab, isatuximab, and MOR202) for treatment of multiple myeloma (Figure 1a) [4,5,6].

The variable domains of conventional antibodies (VH and VL) interact via hydrophobic patches (indicated in black) that help to correctly align the two domains for target binding. The hydrophobic nature of this interaction and its relatively low affinity, however, render scFvs unstable and sticky (Figure 1a). Nanobodies are single variable immunoglobulin domains derived from heavy chain antibodies (hcAbs) that naturally occur in llamas, dromedaries, and other camelids (Figure 1b) [7,8]. The mutation(s) that led to deletion of the CH1 domain in heavy chain antibodies must have occurred >50 million years ago, i.e., in a common ancestor of today’s extant camelids (dromedary, camel, alpaca, llama) [9]. The cluster of V genes in the IgH locus of extant camelids encodes both, highly soluble (VHH) and less soluble (VH) domains. The former occur in the vast majority of camelid hcAbs, the latter in camelid conventional Abs akin to those of mice and men [9]. Camelid VHH domains apparently have been shaped by evolution for high solubility and stability as a single domain. This renders nanobodies particularly suited for reformatting in a LEGO-block like fashion into fusion proteins, including dimers or multimers of nanobodies, chimeric llama/human Nb-hcAbs, biparatopic hcAbs, and Nb-based CARs (Nb-CARs) [10,11]. Dimerization and multimerization of nanobodies has been used to increase the binding strength and the specificity of the constructs [10,11].

**Figure 2:**
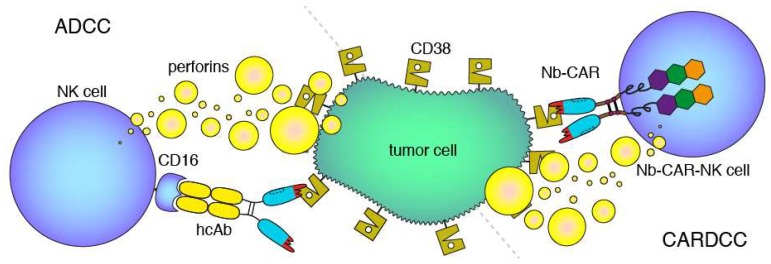
Antibody- and CAR-dependent cellular cytotoxicity. Schematic diagrams of antibody-dependent cellular cytotoxicity (ADCC, left) and chimeric antigen-receptor dependent cellular cytotoxicity (CARDCC, right) mediated by CD38-specific hcAbs and Nb-CARs, respectively. ADCC and CARDCC are mediated by perforins, pore-forming cytolytic proteins released from activated NK cells. Perforins are depicted here schematically as yellow bubbles.

From immunized llamas, we previously isolated a panel of CD38-specific nanobodies [12,13]. We have shown that these nanobodies can be used to construct Nb-hcAbs, some of which exhibit more potent antibody-dependent cellular cytotoxicity (ADCC) than daratumumab (Figure 2) [10,14,15]. 

The aim of the current study was to explore the utility of CD38-specific nanobodies as ligands for Nb-CARs. To this end, we stably transduced the human natural killer cell line NK-92 [16] with CD38-specific and control Nb-CARs. We chose NK-92 cells as effector cells rather than T cells because of their potential use as an “off-the-shelf” reagent. As target cells, we chose established multiple myeloma (LP-1, RPMI-8226) and Burkitt lymphoma cell lines (CA-46, Daudi) with known high cell surface levels of CD38. With these cells, we established CAR-dependent cellular cytotoxicity assays (CARDCC) (Figure 2), using luminescence and flow-cytometry to monitor the killing of target cells (see below). Our results indicate that Nb-CARs, indeed, may provide a basis for clinical development of novel therapeutics to target CD38-expressing tumor cells.

## 2. Materials and Methods

### 2.1. Cell Lines

The following human cell lines were obtained from the German Collection of Microorganisms and Cell Culture (DSMZ, Braunschweig, Germany): NK-92 (natural killer cell line), Daudi and CA-46 (Burkitt lymphoma cell lines), LP-1 and RPMI-8226 (myeloma cell lines). Cell lines (CA-46 luc, Daudi luc, LP-1 luc) stably expressing the luc2 variant of Photinus pyralis luciferase (Promega, Madison, WI, USA) under control of the spleen-focus-forming virus U3 region (SFFV promoter) were generated by lentiviral transduction as described [17,18]. Transduced cells were selected in culture medium containing 1 µg/mL puromycin and subsequently sorted by FACS based on eGFP expression (on a FACS Aria III, BD Biosciences, Heidelberg, Germany). Sorted cells were kept in culture and luciferase-expression was controlled regularly following addition of luciferin using a luminometric plate reader.

As specificity controls, we generated CD38-deficient variants of LP-1 luc, CA-46 luc, Daudi luc, and NK-92. In order to prevent expression of CD38, rather than to merely reduce its expression, we used CRISPR/Cas9 technology (sc-401117-NIC, Santa Cruz Biotechnology, Dallas, TX, USA) rather than shRNA technology. Cells were stained for CD38 using the AlexaFluor647-conjugated JK36 nanobody (JK36^AF647^) and cells were FACS-sorted for lack of cell surface CD38. The sorted cells were maintained in culture and CD38 cell surface levels were controlled regularly using JK36^AF647^.

### 2.2. Generation of Nb-CARs

The human CD38-specific nanobodies WF211, MU1067, JK36 and the ARTC2.2-specific control nanobody s-14 were generated from immunized llamas as described previously [13,19]. In the text, we use the full designation for these nanobodies. For the sake of clarity, we use abbreviated designations for Nb-CARs, e.g., Nb1067-CAR and Nb14-CAR instead of NbMU1067-CAR and Nbs-14-CAR. The nanobody coding region was fused by gene synthesis downstream of the signal sequence of VH and upstream of a strep-tag, the hinge region of human IgG4, the transmembrane and membrane-proximal ITAM domains of human CD28, the ITAM domain of human 4-1BB, and the C-terminal signaling domain of CD3ζ. This cassette was cloned into the gamma-retroviral vector pRSF91.iB.pre* (a derivative of pRSF91.GFP.pre* [20]) upstream of an EMCV internal ribosomal entry site (IRES) and the coding region for blue-fluorescent protein (mTagBFP). HEK293 cells were transiently transfected with the combination of the pRSF91.Nb-CAR.iB.pre* coding for the CAR and packaging plasmids pcDNA3.MLVgp [21] and phCMV-GALV_C4070A_-env to obtain cell-free supernatants containing retroviral particles encoding Nb-CARs. Vector titers were then determined on HEK293T cells as described previously [18].

### 2.3. Stable Transduction of NK-92 Cells with Nb-CARs

NK-92 cells stably expressing Nb-CARs were generated by retroviral transduction. Transduction was carried out in a 24-well plate with 50,000 cells in 500 µL medium per well by addition of 300 µL viral particle-containing supernatant in the presence of 8 µg/mL hexadimethrine bromide and subsequent spin-inoculation for 1 h at 1000× *g* and 25 °C. Stably transduced cells were FACS-sorted based on mTagBFP-expression. CAR-expression by these cells was controlled regularly by staining of cells with AlexaFluor647-conjugated recombinant ectodomains of CD38 and ARTC2.2. The initial transduction efficiency was below 30%; cell sorting resulted in stable expression of the Nb-CAR by more than 95% of cells. The fluorochrome-conjugated ecto-domains of CD38 and ARTC2.2 served as both, positive and negative quality controls for determining the cell surface levels of target-specific Nb-CARs. 

### 2.4. Production of Alexa Fluor 647-Labeled CD38 and ARTC2.2

The myc-his-tagged extracellular domains of CD38 (aa46–300) and ARTC2.2 (aa20–261) were produced in transiently transfected HEK-6E cells cultivated in serum-free medium. Six days post transfection supernatants were harvested and cleared by centrifugation. The myc-his-tagged proteins were purified by immobilized metal affinity chromatography using Ni-NTA agarose (Sigma, St Louis, MO, USA). Fluorochrome-labelling was performed using NHS esters according to the manufacturer’s instructions (Alexa Fluor 647 Succinimidyl Ester, Invitrogen, Karlsbad, CA, USA).

### 2.5. Luminescence CARDCC Assays

CA-46 luc, Daudi luc, and LP-1 luc cells were co-incubated with NK-92-CAR for 4 h at 37 °C at the indicated ratios in αMEM culture medium supplemented with 10% fetal calf serum (FCS), 10% horse serum, 5 mM glutamine, and 5 ng/mL interleukin 2 (IL-2 Proleukin-S, Novartis, Basel, Switzerland). D-luciferin (Biosynth, Staad, Switzerland) was added as substrate (75 µg/mL) for 20 min and bioluminescence-intensity (BLI) was measured with a microplate reader (Victor^3^, Perkin Elmer, Boston, MA, USA). 

### 2.6. Flow Cytometric CARDCC Assays

Target cells were fluorescently pre-labeled by incubation with AlexaFluor647, effector cells by incubation with eFluor450. Cells were washed and co-incubated at the indicated E:T-ratios at 37 °C for the indicated time-periods. Dead cells were stained with propidium iodide (PI, Invitrogen, WA, USA) or Pacific Orange succinimidyl ester (PacO, Thermo-Fisher Scientific, Waltham, MA, USA) before analysis of cells by flow cytometry (BD FACS Celesta/Becton Dickinson). Percentage of cells was calculated as follows: % lysis [%] = 1 − (cells [sample]/ cells [sample with control CAR]) × 100%.

### 2.7. CARDCC Assays with Primary Human Bone Marrow Samples

Fresh bone marrow aspirates were obtained from patients after Institutional Review-Board-approved consent (PV5505). Bone marrow mononuclear cells (BM-MNCs) were prepared by Ficoll-Paque density gradient centrifugation of bone marrow aspirates and subsequent depletion of remaining erythrocytes using red blood cell lysis buffer (NH4Cl + KHCO3 + EDTA). BM-MNCs were co-incubated with eFluor450-labeled NK-92 Nb-CAR cells at an effector to target ratio [E:T] of 1:1 for 4 h at 37 °C in αMEM culture medium (see above). Cells were then stained with a panel of fluorochrome-conjugated antibodies (CD38, CD45, CD138/229, CD269/CD319/CD56, CD19) and PacO and analyzed via flow cytometry. We did not use CD138 in these four hour assays because of the known instability of this marker on the cell surface of MM cells [22]. Staining of CD38 was achieved with Alexa Fluor 647-conjugated nanobodies that bind independently of the nanobody contained in the CAR: JK36^AF647^ or MU523^AF647^ for Nb211-CAR, MU523^AF647^ or WF211^AF647^ for Nb36-CAR, and JK36^AF647^ or WF211^AF647^ for Nb1067-CAR. An FSC threshold was set to exclude debris while including the population of small CD19^+^ B cells. NK-92 cells and dead cells were excluded via staining by eFluor450 and Pacific Orange, respectively. MM cells were identified by high co-expression of CD38 and CD56 or CD319. Numbers of MM cells were determined using CountBright absolute counting beads (Invitrogen, Karlsbad, CA, USA). Percentage of surviving MM cells was calculated as follows: Percent of survival [%] = (MM cell number per µL [NK-92-CAR-treated sample]/MM cell number per µL [untreated sample]) × 100%. Significance between CD38-specific Nb-CAR-NK and the control Nb-CAR-NK was calculated using unpaired T-test (GraphPad Prism, GraphPad Software, CA, USA).

## 3. Results

### 3.1. Generation of CD38-Deficient Cell Lines and Lentiviral Transduction of CD38^+^ and CD38^−^ Cell Lines with Luciferase

**Figure 3:**
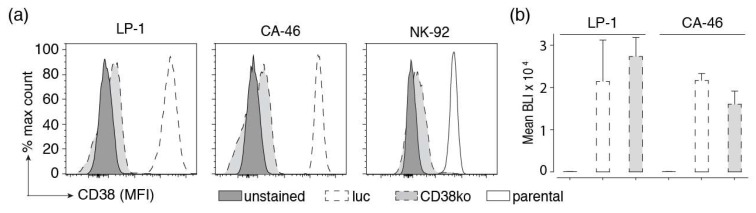
Inactivation of CD38 and stable expression of luciferase in established cell lines. (**a**) The CD38 gene was inactivated in the indicated tumor cell lines using CRISPR/Cas9 technology to provide specificity controls. Cells were FACS-sorted for lack of CD38 cell surface expression. Parental and CD38ko cells were incubated for 30 min at 4 °C with Alexa Fluor 647-conjugated Nb JK36 before analysis by flow cytometry. Results are representative for three similar experiments. (**b**) LP-1 and CA-46 cells and their corresponding CD38ko cell lines were lentivirally transduced with luc-GFP, encoding luciferase and GFP separated by an internal ribosomal entry site. GFP was used for cell sorting, luciferase was used for monitoring cell vitality by bio-luminometry. Cells were FACS-sorted for high expression of GFP. Triplicate samples of parental luc-GFP-expressing cells were incubated in a 96 well plate for 20 min at 24 °C with luciferin before analysis for luminescence with a plate reader. Results are representative for two similar experiments.

CD38 is overexpressed by several established human tumor cell lines, including LP-1 multiple myeloma, CA-46 and Daudi Burkitt lymphoma, and NK-92 natural killer cell lymphoma [1,2,3]. As specificity controls, we inactivated the CD38 gene in these cell lines using CRISPR/Cas9 technology. Cells were monitored for cell surface expression of CD38 using Alexa Fluor 647-conjugated CD38-specific nanobodies and CD38-deficient cells were FACS sorted for lack of CD38 cell surface expression (Figure 3a). The results confirm high expression of CD38 by parental cells and lack of CD38 expression by CD38ko cells. Similar results were obtained with Daudi luc cells (not shown).

To permit luminescence-based cellular cytotoxicity assays, we stably transduced LP-1, CA-46, and Daudi cells with firefly luciferase and GFP. Cells were FACS sorted for high levels of GFP expression. Cells were monitored for luciferase activity following addition of luciferin using a luminescence plate reader. The results show lack of detectable bioluminescence by parental cells and very high levels of BLI signals in all luciferase-transduced cell lines (CD38^+^ and CD38ko cells) (Figure 3b). Killing assays revealed a high dynamic range of BLI signals in all luciferase-transduced cell lines (see below). Similar results were obtained with Daudi luc cells (not shown).

### 3.2. Generation of Nanobody-Based Chimeric Antigen Receptors (Nb-CARs) and Transduction of NK-92 Cells with Nb-CARs

**Figure 4:**
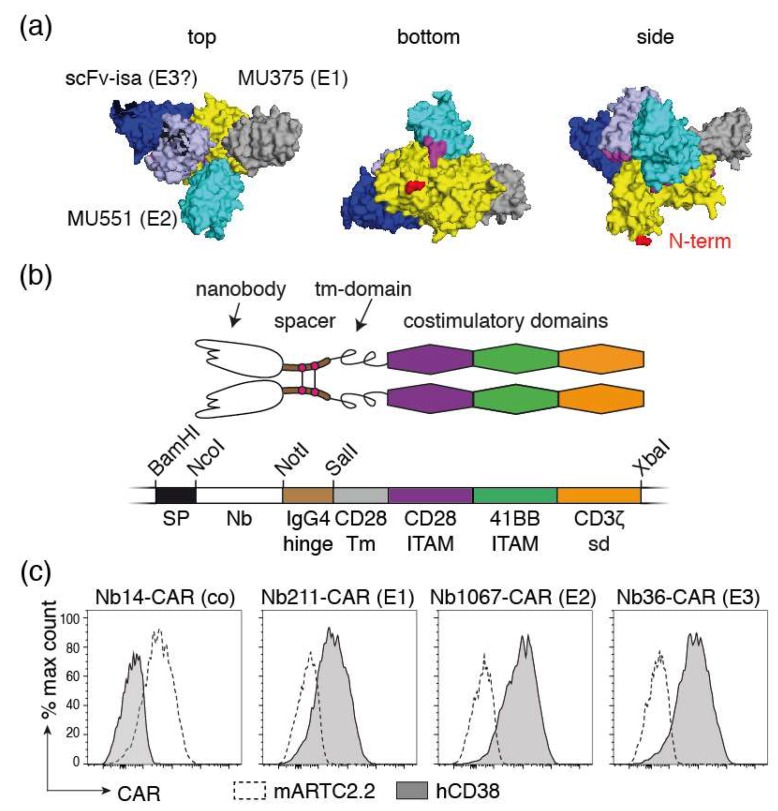
NK-92 cells stably expressing Nb-CARs. (**a**) 3D model of the extracellular domain of CD38 (yellow) in complex with two nanobodies (MU375 grey, and MU551 cyan) and the scFv of isatuximab (VH and VL in dark and light blue, respectively). The N-terminal amino acid of CD38 (depicted in red) connects to the membrane proximal amino acids and transmembrane domain. The model is presented from three points of view: the putative membrane-proximal side is designated “bottom,” the membrane distal side “top” and the putative view parallel to the plane of the cell membrane “side.” WF211 binds to the same epitope as MU375 (designated E1), MU1067 to the same epitope as MU551 (E2), and JK36 to a third epitope (E3). It is likely, but not yet confirmed experimentally, that JK36 and isatuximab bind to a similar epitope. (**b**) Schematic diagram of a Nb-CAR. The coding region for the Nb-CAR was assembled by gene synthesis. The indicated restriction enzyme recognition sites were incorporated to allow flexible exchange of the corresponding elements. SP: signal peptide; Nb: nanobody, tm: transmembrane; ITAM immunoreceptor tyrosine-based activation motif, sd: signaling domain. (**c**) NK-92 cells were stably transduced with the indicated Nb-CARs and sorted for high expression of blue fluorescent protein BFP, which was encoded downstream of an IRES sequence behind the coding region for the Nb-CAR. Cells were incubated with the fluorochrome-conjugated recombinant extracellular domains of mouse ARTC2.2 or human CD38 before analysis by flow cytometry. Results are representative for three similar experiments.

Nanobodies WF211, MU1067, and JK36 bind to three distinct, non-overlapping epitopes on CD38, designated E1, E2, and E3, respectively [13]. MU1067 inhibits the enzymatic activity of CD38, while WF211 and JK36 do not inhibit or enhance its activity [13]. The respective epitopes can be inferred from published crystal structures of CD38 in complex with nanobodies MU375 and MU551 and the Fab fragment of isatuximab (Figure 4a). WF211 competes for binding to CD38 with MU551 (E1), MU1067 competes for binding with MU375 (E2). JK36 binds to a third, non-overlapping epitope (designated E3) and competes neither with WF211 nor with MU1067 for binding to CD38. The Fab fragment of isatuximab also binds to an epitope that does not overlap with epitopes E1 and E2 (Figure 4a). It is possible, but not yet established experimentally that JK36 binds to a similar epitope as isatuximab. 

For construction of Nb-CARs, we fused each of the three CD38-specific nanobodies and a control nanobody (the ARTC2.2-specific nanobody s-14) genetically to the components of a classic third-generation CAR, i.e., the hinge region of IgG4, the transmembrane and intracellular ITAM domains of CD28, and the cytosolic signaling domains of 4-1BB and CD3ζ (Figure 4b). We cloned these Nb-CAR encoding cassettes into a retroviral vector upstream of an IRES followed by the coding region for blue fluorescent protein (BFP). NK-92-CD38ko cells were stably transduced with these vectors and cells were sorted for high levels BFP expression and for high levels of cell surface Nb-CAR.

In order to detect cell surface levels of Nb-CARs, we monitored cell surface levels of the respective Nb-CARs by flow cytometry using the soluble ecto-domains of CD38 and ARTC2.2 conjugated to AlexaFluor647 (Figure 4c). The results show that CD38^647^ specifically binds to NK-92 cells transduced with CD38-specific Nb-CARs but not to cells transduced with the ARTC2.2-specific Nb-CAR, whereas ARTC2.2^647^ binds to NK-92 cells transduced with the ARTC2.2-specific Nb-CAR but not to NK-92 cells transduced with any of the three CD38-specific Nb-CARs. 

### 3.3. Luminescence-Based CAR-Dependent Cellular Cytotoxicity Assay (CARDCC)

**Figure 5:**
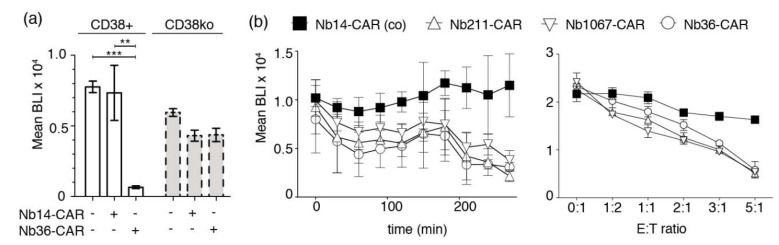
Luminescence-based CARDCC assay of CD38-expressing tumor cells. (**a**) CD38^+^ or CD38ko CA-46 cells stably transduced with luciferase were incubated for 4 h at 37 °C in the absence (−) or presence (+) of NK-92 cells expressing either the control Nb14-CAR or the CD38-specific Nb36-CAR at an effector-to-target ratio of 4:1. Bioluminescence signal intensities (BLI) were measured 20 min after addition of luciferin. Significance between CD38-specific and control Nb-CAR-NK cells was calculated using unpaired T-test (**, *p* < 0.01; ***, *p* < 0.005). (**b**) The left and right panels show kinetic and dose response analyses of CARDCC toward luciferase-expressing CA-46 cells. Left panel: Nb-CAR-NK cells were co-incubated with CA-46 cells for the indicated times at 37 °C at an effector-to-target ratio of 1:1 before addition of luciferin and bio-luminometric analysis. Right panel: Nb-CAR-NK cells were co-incubated with CA-46 cells for 4 h at 37 °C at the indicated effector-to-target (E:T) ratios before bioluminometry. Results are representative for three similar experiments.

In order to test whether Nb-CAR-NK cells specifically lyse CD38-expressing tumor cells, we co-incubated Nb-CAR-NK cells with luciferase-transduced CA-46 cells for 4 h at 37 °C before addition of luciferin. Luciferase activity was then monitored with a luminescence plate reader (Figure 5a). The results show that CD38-specific Nb-CAR-NKs effectively lyse CD38-expressing tumor cells but not the respective CD38ko daughter cell line. Time course analyses revealed that CARDCC happens in a time-dependent manner (Figure 5b). Similarly, the results of titration analyses show that CARDCC occurs in a dose-dependent manner.

### 3.4. A Flow-Cytometric CARDCC

**Figure 6:**
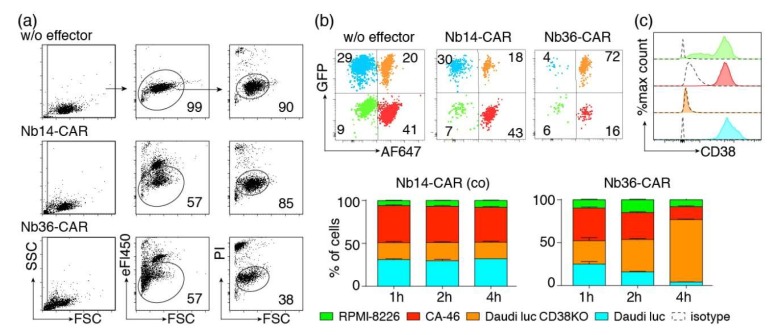
Flow-cytometric CARDCC assay of CD38-expressing tumor cells. (**a**) CA-46 cells were co-incubated for 4 h at 37 °C without (w/o) or with eFluor450-labeled NK-92 cells expressing either the control Nb14-CAR or the CD38-specific Nb36-CAR in an effector to target ratio of 1:1. Cells were analyzed by flow cytometry 10 min after addition of propidium iodide. Numbers indicate the percentage of cells in the gated population. (**b**) A mixed population of four color-coded cell lines was incubated for 1–4 h at 37 °C with or without (w/o) eFluor450-labeled Nb-CAR-NK cells at an effector-to-target ratio of 1:1. CD38^lo^ RPMI-8226 cells were unlabeled. CD38^hi^ CA-46 cells were pre-labeled with Alexa Fluor 647. CD38^+^ and CD38ko Daudi luc cells were GFP positive, CD38ko Daudi cells were pre-labeled with AlexaFluor647. Gating was performed on live (PI-negative), target (eF450-negative) cells. (**c**) To detect cell surface levels of CD38, the target cells were stained with AlexaFluor647-conjugated MU1067-hcAb and with an AlexaFluor647-conjugated control hcAb (dashed lines). Color coding of cells is as in (b). Results are representative for three similar experiments.

We next set out to monitor CARDCC by flow cytometry. As a means to distinguish target from effector cells, we labeled Nb-CAR-NK cells with the fluorescent dye eFluor450. To monitor cell death, we used the DNA staining dye propidium iodide, which is excluded by living cells. We co-incubated eFluor450-labeled Nb-CAR-NK cells with CA-46 cells for 4 h at 37 °C before addition of propidium iodide and flow-cytometric analysis (Figure 6a). The results confirm that NK cells expressing the CD38-specific Nb36-CAR, but not those expressing the control Nb14-CAR, indeed effectively lyse CD38-expressing tumor cells. CARDCC caused both—staining by propidium iodide and a decrease in forward light scatter (FSC). The latter likely reflects the shrinkage of lysed cells. 

We next set out to determine whether Nb-CAR-NK cells could specifically deplete CD38-expressing cells in mixed populations of CD38^+^ and CD38^−^ cells. To this end we co-incubated CD38^+^ and CD38ko Daudi cells, CD38hi CA-46 cells and CD38^lo^ RPMI-8226 cells for 1–4 h with eFluor450-labeled Nb-CAR-NK cells (Figure 6b). The four target cell lines were distinguished on the basis of GFP expression (Daudi luc and Daudi luc CD38ko) and pre-labeling of cells with Alexa Fluor 647 (CA-46 and Daudi luc cells). The results show that CD38-specific but not control CAR-NK cells specifically deplete CD38 expressing cells. 

### 3.5. CD38-Directed Nb-CAR-NKs Specifically Deplete CD38^+^/CD56^+^ Myeloma Cells from Primary Human Bone Marrow Samples

**Figure 7:**
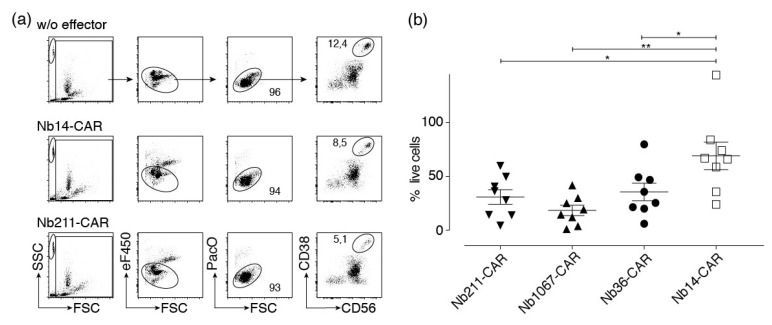
CD38-specific Nb-CAR-NKs deplete CD38^+^ myeloma cells. Primary human bone marrow cells were co-incubated for 4 h at 37 °C without (w/o) or with eFluor450-labeled NK-92 cells expressing either a control CAR (Nb14) or a CD38-specific CAR (Nb211) in an effector to target ratio of 1:1. Cells were counterstained with fluorochrome conjugated αCD56, αCD319, and a CD38-specific Nb that binds a non-overlapping epitope to that of the Nb contained in the CAR before analysis by flow cytometry. (**a**) Beads (SSC^hi^/FSC^lo^), effector cells (eF450^+^), and dead cells (PacO^hi^/FSC^lo^) were excluded by gating. Numbers indicate the percentage of cells in the gated population. Results are representative for eight similar experiments (one for each of eight patient samples). (**b**) The number of surviving myeloma cells (CD38^+^/CD56^+^ or CD38^+^/CD319^+^) were determined with the aid of cell counting beads. Percentages of surviving myeloma cells were calculated relative to the number of surviving myeloma cells in the absence of effector cells (set at 100%). Gating was performed as in (**a**). Significance between CD38-specific and control Nb-CAR-NK cells was calculated using unpaired T-test (*, *p* < 0.05; **, *p* < 0.01)

In the final set of experiments we assessed CARDCC against primary multiple myeloma cells from bone marrow samples of eight human myeloma patients. Myeloma cells were identified by their high levels of cell surface CD38 (and CD56 or CD319). The percentage of such cells in the patient samples ranged from 1.4 to 16.9% (mean 7.1%). We excluded dead cells on the basis of low FSC and staining by PacO. Effector Nb-CAR-NK cells were excluded by staining with eFluor450. In order to permit quantification of absolute cell numbers we added counting beads to the samples (Figure 7a). We found that incubation of bone marrow samples with CD38-specific Nb-CAR-NK cells mediates a significantly higher loss of myeloma cells than incubation with control Nb-CAR-NK cells (Figure 7b). 

## 4. Discussion

Our results demonstrate that CD38-specific nanobodies provide effective ligands for chimeric antigen receptors. We show that the established human NK-92 cell line can be retrovirally transduced to stably express nanobody-based CARs. CAR-expressing NK cells, as a permanent cell line, have the potential to serve as an “off-the-shelf” reagent [16,23]. NK-92 cells were originally isolated from the peripheral blood of a 50-year-old patient with non-Hodgkin Lymphoma in 1992 [24]. These cells resemble the phenotype of an activated NK cell. In phase-I trials, NK-92 cells were found to have a high safety profile and to induce only mild graft-versus-host-disease [25,26]. 

Our results support previous studies demonstrating the potential of nanobody-based CARs [27,28,29,30]. Here we used CARs constructed from nanobodies directed against three different epitopes of CD38. All of these mediated effective CARDCC indicate that these epitopes of CD38 are accessible to the CARs. The results further suggest that there is not a preferred epitope of CD38 for CAR-mediated cytotoxicity.

**Figure 8:**
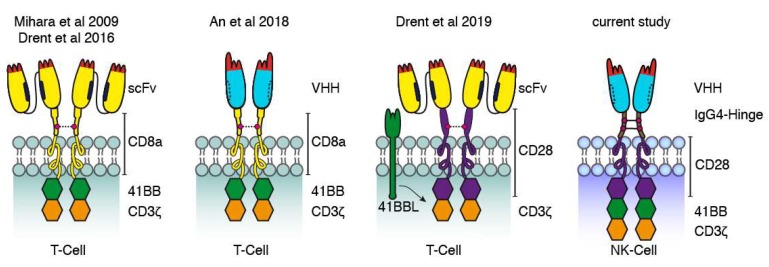
Comparison of CD38-specific CARs used in the current and previous studies. Schematic diagrams illustrating the structures of CD38-specific conventional and Nb-based CARs used in this study to transduce NK-92 cells and in previous studies to transduce T cells. The first conventional and Nb-based CARs reported consisted of a CD38-specific scFv or Nb connected via the hinge and transmembrane domains of CD8a to the signal transduction domains of 4-1BB and CD3ζ [31,32]. Recently, a more effective conventional CAR was reported carrying the hinge, transmembrane, and signal transduction domains of CD28 and a separate expression-cassette for 4-1BBL transduced into T cells. The Nb-CAR of the current study carries the IgG4 hinge and three cytosolic signal transduction domains.

Our data are also in line with those of previous studies using CD38-specific CARs based on scFvs and Nbs (Figure 8). Mihara et al. and Drent et al. fused the single-chain variable fragment of CD38-specific mAbs via the hinge and transmembrane domains of CD8a to the signal transduction domains of 4-1BB and CD3ζ [31,32]. An et al. used a similar architecture for a nanobody-based CAR [27]. More recently, Drent et al. replaced the 4-1BB costimulatory domain with a CD28-costimulatory domain and co-expressed 4-1BBL to provide additional 4-1BB signaling [33]. Our CAR-constructs in turn use the IgG4 hinge and the CD28 transmembrane region as a bridge to the signal transduction domains of CD28, 4-1BB, and CD3ζ. In all cases, cysteine residues in the extracellular linker likely mediated the formation of covalently linked dimers. It remains to be determined whether the efficacy of these constructs can be enhanced further by adjusting the length of the extracellular stalk, preventing disulfide formation, e.g., by site directed mutagenesis of cysteine to serine, and/or by replacing the signal transduction modules derived from the T-cell surface proteins with signal transduction modules derived from NK cell surface proteins. 

The interaction between CAR-expressing effector cells and target cells expressing the cognate antigen presumably involves multivalent binding, i.e., the interaction of effector and target cells might encompass the simultaneous engagement of many CARs and cognate antigens on the respective surfaces of effector and target cells. Because non-tumor cells also express CD38, it would be beneficial for cancer therapy if Nb-CAR-NK cells would preferentially deplete CD38^high^ cells. We found that RPMI cells with slightly lower levels of CD38 were more resistant to CARDCC after 2 h of co-incubation with Nb-CAR-NK cells. However, we cannot exclude that other cell surface proteins such as MHCI or inhibitory NK cell receptors influence the susceptibility to CARDCC.

The affinity of the ligand-binding domain of the CAR may also influence the relative cytotoxicity of CAR-expressing cells to target cells that express different levels of the target antigen [34,35,36]. A low-affinity CAR may preferentially direct CAR-expressing T cells or NK cell to tumor cells that express high surface levels of the target antigen, while sparing normal cells that express lower levels of the target antigen, thereby reducing the potential off target toxicity of CAR-T and CAR-NK cells. Since nanobodies consist of only a single immunoglobulin domain, it will likely be technically easier to modulate the affinity of a nanobody-based CAR than that of a scFv-based CAR [7,8,11]. 

The specificity may be enhanced by co-targeting of distinct targets, i.e., to two membrane proteins that are co-expressed on tumor cells [37]. This would reduce the unwanted binding of CAR-T or CAR-NK cells to normal cells that express only one of these two target proteins. Based on their excellent solubility and reformating capacity, nanobodies may prove more suitable than conventional scFvs for such approaches [38].

## 5. Conclusions

Nanobody-based chimeric antigen receptors (Nb-CARs) may provide a basis for the clinical development of novel therapeutics to target CD38-expressing tumor cells.
